# Non-informative vision improves spatial tactile discrimination on the shoulder but does not influence detection sensitivity

**DOI:** 10.1007/s00221-020-05944-2

**Published:** 2020-10-13

**Authors:** Fabrizio Leo, Sara Nataletti, Luca Brayda

**Affiliations:** 1 Robotics, Brain, and Cognitive Sciences, Istituto Italiano di Tecnologia, Genoa, Italy; 2 DIBRIS, University of Genoa, Genoa, Italy; 3 Acoesis s.r.l, Genoa, Italy

**Keywords:** Tactile acuity, Numerosity judgment, Tactile sensitivity, Visuo-tactile, Somatosensory cortex, Visual enhancement of touch

## Abstract

Vision of the body has been reported to improve tactile acuity even when vision is not informative about the actual tactile stimulation. However, it is currently unclear whether this effect is limited to body parts such as hand, forearm or foot that can be normally viewed, or it also generalizes to body locations, such as the shoulder, that are rarely before our own eyes. In this study, subjects consecutively performed a detection threshold task and a numerosity judgment task of tactile stimuli on the shoulder. Meanwhile, they watched either a real-time video showing their shoulder or simply a fixation cross as control condition. We show that non-informative vision improves tactile numerosity judgment which might involve tactile acuity, but not tactile sensitivity. Furthermore, the improvement in tactile accuracy modulated by vision seems to be due to an enhanced ability in discriminating the number of adjacent active electrodes. These results are consistent with the view that bimodal visuotactile neurons sharp tactile receptive fields in an early somatosensory map, probably via top-down modulation of lateral inhibition.

## Introduction

A consistent body of knowledge has shown that vision can influence touch. For instance, the visual enhancement of touch (VET) effect is the facilitation in spatial acuity we observe when we see the body part being touched without seeing the actual tactile stimulation (Kennett et al. 2001; see, for a review, Eads et al. 2015). While most studies used an object placed in the same position as the body part as a control condition, other studies instead simply occluded the body part from view (e.g. Harris et al. 2007; Catley et al. 2014). Interestingly, the VET does not require proprioceptive orienting towards the stimulated body part, as the participants showed visual enhancement of touch also when they observed the stimulated body part through a monitor (Tipper et al. 1998) or even another person’s body part (Haggard 2006; Beck et al. 2015).

Most of the studies investigating the VET effect have tested body locations that can be normally seen by the subjects, in particular the hand and the upper limb but also the foot (Serino et al. 2009). These body parts can be easily the object of visual attention. They are daily before our eyes. It is plausible that these locations, involved also in exploratory movements, are subserved by bimodal visuo-tactile neurons already observed in monkey brain (Graziano and Gross 1993; Zhou and Fuster 2000). The presence of the VET in body parts normally not viewed and less linked to exploratory movements has been tested more rarely and the findings seem to be contradictory. For instance, Tipper et al. (2001) found evidence of VET on the neck, although the effect was smaller than that observed at the more visually familiar face. However, the authors did not measure spatial acuity but only a response time (RT) facilitation when participants had concurrent vision of the stimulated body location on a monitor. More importantly, their experiments did not completely exclude spatial attention components. Participants indeed attended to tactile events in any of several body locations while only one body location was viewed on the monitor. It is likely that in these conditions of uncertainty, viewing a specific body part might have increased attention to that body part compared to the others. More recently, Catley et al. (2014) investigated the same issue with the back as body location. Their results showed evidence of VET at the back only in one out of three experiments. The authors concluded that seeing the back does not enhance tactile acuity and the absence of the effect might indicate that there are no bimodal neurons in humans subserving this body area. Alternatively, it is possible that the shrunken view of the back through the monitor might have abolished the effect. On the one hand, Kenneth et al. (2001) have indeed shown how magnifying the visual input could produce a larger VET effect. Therefore, it is likely that shrinking the representation of a body location might diminish the effect of seeing it. On the other hand, Treshi-marie Perera et al. (2015) have shown how shrinking the representation of a finger actually enhances tactile sensitivity. They hypothesized that the effect might be due to the reduction of visual information which resulted in a lower weighting of the visual signal (Ernst and Banks 2002). However, it is possible that the distortion of visual information might influence differently a spatial discrimination task that might be favored by a visual representation spatially similar to the somatotopic representation of the stimulated body part or even more detailed. In summary, it is currently unclear whether non-informative vision improves tactile perception in a body location normally not seen.

We, therefore, investigated whether vision enhances tactile perception in a body location that is usually not viewed. To do so, participants performed a numerosity judgment task of electrotactile stimuli administered on the shoulder while watching a real-time representation of their shoulder. Importantly, unlike Catley et al. study (2014), the image of the shoulder had approximately the same dimension as the real body part. In addition, unlike Tipper et al. (2001), we did not measure only response times, but also enumeration ability and spatial sensitivity. The numerosity judgment task was indeed preceded by a tactile detection threshold estimation task performed while the participants viewed the stimulated body part or not. Most of the previous studies on the VET have indeed only investigated the effect of vision on spatial acuity in tasks such as two-point discrimination (Kennett et al. 2001; Serino et al. 2009; Catley et al. 2014) or grating orientation discrimination (Taylor-Clarke et al. 2004; Cardini et al. 2011, 2012). To our knowledge, only Harris et al. (2007) tested also tactile sensitivity and found reduced discrimination ability at near-threshold levels when viewing the stimulated hand. On the other hand, they did not test body parts which are normally not viewed. In addition, no previous studies tested other, more complex, tactile skills such as the ability to enumerate simultaneous tactile stimuli. In summary, the novelty of our study is threefold: first, we investigate whether the VET occurs in a usually not seen body location. Second, we test the effect of non-informative vision of this body part on numerosity judgment and tactile sensitivity. Third, we manipulate the level of difficulty of the same numerosity judgment task. To do so, we make the assumption that increasing the numerosity of tactile stimuli makes the numerosity judgment more difficult. This assumption is well supported by previous findings showing that the underestimation of the number of stimuli increases with the number of tactile stimuli (Gallace et al. 2006, 2007; Wang et al. 2018).

Our general hypothesis is that numerosity judgment would be improved when participants could view their shoulder as opposed when they could not. This effect might be mediated by bimodal visuotactile neurons. Even though the existence of such neurons with receptive fields on the shoulder has not been directly demonstrated yet, several non-human primate studies have already found these cells in the ventral premotor and posterior parietal cortices (Fogassi et al. 1996; Graziano et al. 1997; Duhamel et al. 1998) and cross-species comparisons of these multimodal brain regions have generally found good correspondence between monkeys and humans (Bremmer et al. 2001; Makin et al. 2007). On the contrary, we hypothesized that vision would not play the same role for tactile sensitivity. If it is true that the visual enhancement of touch is due to a top-down modulation of lateral inhibition (Kennett et al. 2001; Press et al. 2004; Haggard et al. 2007), then the effect should be specific for spatial discrimination tasks and absent for tactile sensitivity tasks. Finally, we expected a stronger visual enhancement of touch with increasing of the level of difficulty since previous studies showed bigger effect of visual feedback when the task was very challenging (e.g. Press et al. 2004).

## Materials and methods

### Participants

Twelve naïve, healthy volunteers (age 25–31, mean 27 ± 2 years, eight females) with no known cognitive or tactile deficits took part in the experiment. The experiment was approved by the Region Liguria Ethical Committee (approval ID 172REG2016, approval date September 13, 2016).

### Experimental setup

**Fig. 1:**
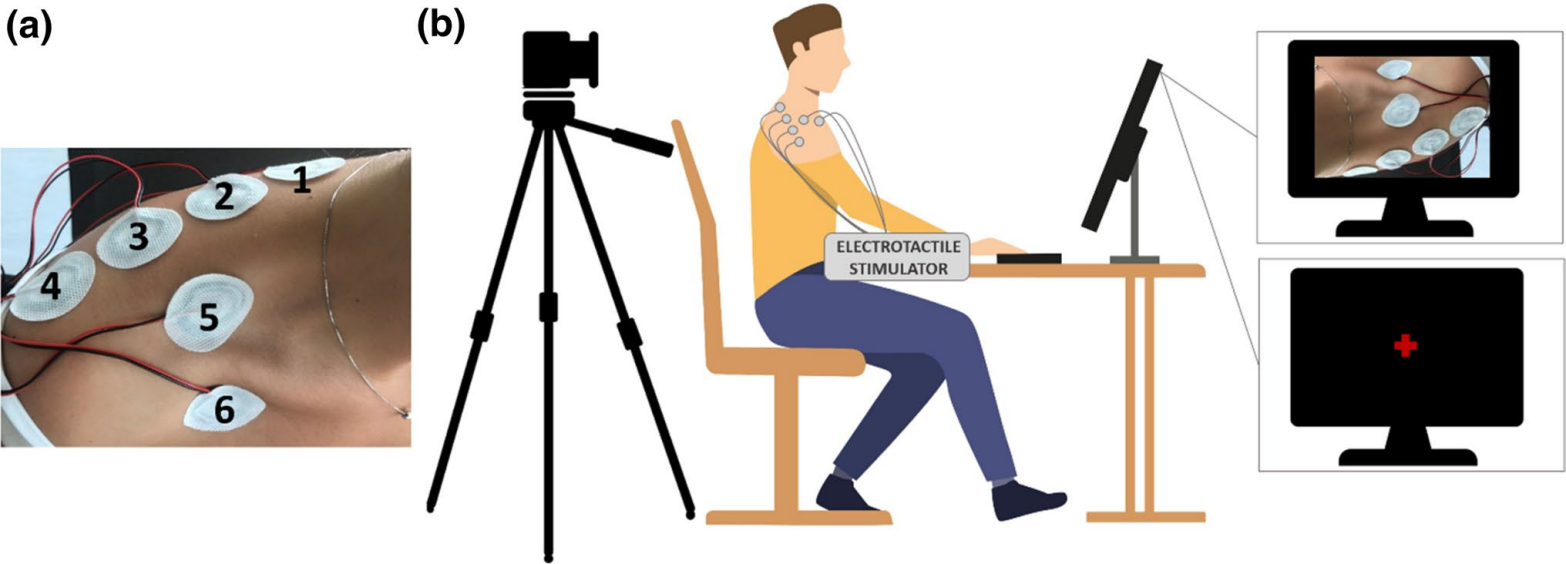
*Left Panel* placement of the electrodes on the participants’ shoulder. *Right panel* experimental setup comprising (i) a standard laptop computer equipped with a Bluetooth low-energy module, (ii) a current-controlled multichannel electrotactile stimulator equipped with six concentric electrodes, (iii) a full HD webcam (Logitec C920). The right part of the figure shows the two different conditions counterbalanced across participants: “view shoulder” (top panel) and “view fixation” (bottom panel) condition

Participants were comfortably seated centrally in an adjustable-height chair in front of a table approximately 60 cm from a computer screen with their hands positioned on a keyboard. A camera (Logitec C920 HD Pro Webcam) was suspended directly above the dominant shoulder of participants, pointing straight down to stream, when necessary, a full-size real-time footage of participants’ shoulder (Fig. 1).

The supplemental tactile stimulation was provided using a current-controlled multichannel electrotactile stimulator prototype WESP (Global Electronics), which incorporates technology for time and space distribution of stimuli introduced by Tecnalia with the IntFES system (Malešević et al. 2012). Six self-adhesive concentric electrodes were placed on the shoulder and upper back with inter-electrode distance well above the two-point discrimination threshold for electrical stimulation on that body location (Solomonow et al. 1977; see also Mancini et al. 2014 for a mechanical measurement). Specifically, four electrodes were distributed equidistantly (5 cm in between) on the backside of the shoulder along a horizontal line from the base of the neck to the end of the shoulder and two on the front side (one above and one below the collarbone, see Fig. 1).

The stimulation parameters could be set online by sending simple text commands to the stimulator. The stimulator was interfaced via Bluetooth to a portable laptop computer running a custom script within the MATLAB R2018a computing environment (MathWorks Inc., Natick MA) plus Psychtoolbox (Brainard 1997; Kleiner et al. 2007). Pulse width (300 µs), frequency (100 Hz) and duration of the stimulation (200 ms) were kept constant. We selected 100 Hz as stimulation frequency since it has been shown to elicit a well-localized and continuous sensation resembling constant pressure (Chai et al. 2013, 2015; Wang et al. 2013; D’Alonzo et al. 2014; Zhang et al. 2015; Štrbac et al. 2016). Frequencies below 100 Hz were not taken into consideration because they produce a vibration feeling and higher frequencies elicit a fused tingling sensation. On the contrary, the stimulation intensity was adjusted for each subject individually based on his/her detection threshold (see “Experimental procedure”).

### Experimental procedure

**Fig. 2:**
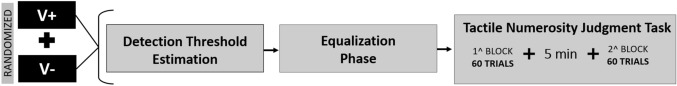
Flow chart of the experimental procedure. Subjects performed two randomized sessions (one for each condition: V + and V−). Each session included three phases: threshold estimation phase, equalization phase and tactile numerosity judgment task

Figure 2 shows an outline of the experimental procedure. Each participant took part in two sessions, one for each experimental condition. Two different experimental conditions were implemented in this study:

”View shoulder” (V +): participants had to look at the screen showing real-time footage of their shoulder;

”View fixation” (V−): participants had to look at a fixation cross displayed at the center of a computer screen.

The two conditions were consecutively performed on the same day, separated by a few minutes break. The order of the two conditions was counterbalanced across participants to minimize training effects. Each session lasted about 40 min and comprised three phases: detection threshold estimation, equalization and tactile numerosity judgment task.

### Detection threshold estimation

First, we estimated the detection threshold (DT) for the electrode 1 (see Fig. 1) using a 1-up and 1-down staircase procedure, where the current amplitude was changed trial by trial according to the subject’s response. In this phase, participants were asked to report the presence or absence of the electrical stimulus by a verbal ‘yes’ or ‘no’. Starting from a subthreshold current amplitude (0.5 mA), we automatically increased the amplitude, with a step-size of 0.1 mA, until the subject reported the presence of the stimulus. Whenever the participant detected the stimulus, the current’s intensity decreased; whenever, the participant did not detect the stimulus, the current’s intensity increased by the same step size. The points at which the subject response changed direction, i.e. response reversals, were recorded. The staircase procedure was run until the participant completed six reversals, and the detection threshold was taken as the average amplitude of the amplitude values corresponding to the last four reversals. Finally, the amplitude for electrode 1 was set to 3 times the DT and kept constant during the experiments. This amplitude was chosen to elicit a clear, comfortable and well-localized sensation and was also adopted as the reference stimulus (RS) for electrode 1 in the first stage of the following Equalization phase.

### Equalization phase

To avoid the possibility of participants making discriminatory judgments based on intensity rather than spatial position, the stimulation intensities across the six electrodes were equalized using five 2-interval forced-choice (2IFC) tasks. In each 2IFC task, a pair of stimuli—the RS at one electrode (RS*n*, where *n *= 1, …, 5 indicates the electrode number) and the test stimulus at the adjacent electrode (*n *+ 1)—were presented. The two stimuli (0.2–s long) were delivered one at a time in two successive intervals with interstimulus interval (ISI) = 1 s, and the order of presentation varied randomly from trial to trial. While the RS amplitude was kept constant, the amplitude of the test stimulus varied from trial to trial. The test stimulus was initially set equal to a third of the RS, and was increased or decreased in steps of 0.1 mA depending on participants’ response. In each trial, participants were asked to indicate which was the strongest stimulus, the first or the second perceived. As in the DT estimation phase, the procedure was run until the participant completed six reversals. This task was performed five times iteratively for pairs of adjacent electrodes, one for each electrode whose amplitude had to be determined. Therefore, the RS number *n* and the corresponding adjacent electrode (*n *+ 1) changed as a function of the number *n* of the 2IFC task (*n* from 1 to 5). In the first 2IFC, the RS was the electrode 1 (whose amplitude was already determined in the DT phase) and the test stimulus was the electrode 2 (whose intensity had to be determined). In the second 2IFC, the RS was the electrode 2 (whose amplitude was just been determined) and the test stimulus was the one presented to the electrode number 3 (to be determined) and so on. As a last step, the experimenter activated the six electrodes in sequence and, if the participant experienced different intensities across electrodes, small adjustments in amplitudes were made to make them equal.

### Tactile numerosity judgment task

After the equalization phase, participants performed a tactile numerosity judgment task. This phase lasted about 20/30 min and comprised two blocks of 60 trials each with a 5-min break between blocks. In each trial, a random combination of electrodes was activated simultaneously for 200 ms. For each number of electrodes (from 1 to 6), different activation patterns were chosen randomly among all the possible combinations. Participants were asked to keep looking at the screen showing either their shoulder or a fixation cross and to press the numeral key (from 1 to 6) on a keyboard corresponding to the number of perceived electrodes. They were also asked to respond as accurately and as fast as they could but with a stronger emphasis on accuracy. Each number of activated electrodes (1 to 6) was presented for 20 times giving rise to a total of 120 trials.

### Data analysis

We collected the following dependent variables for each condition: (1) tactile detection threshold, (2) numerosity judgment accuracy, (3) signed error and (4) response time. Detection threshold was defined as the level of intensity of the stimulus which allowed detection 50% of the time. Numerosity judgment accuracy was defined as the percent success rate in identifying the number of presented stimuli. Signed error was defined as the difference, in terms of the number of electrodes, between the participant’s response and the correct answer allowing us to identify potential bias in estimating the number of electrodes (e.g., over/underestimation).

We assessed the normality of the outcomes’ distribution using the Shapiro–Wilk test. As the test showed that most of the data were not normally distributed, we used non-parametric tests for statistical analysis.

First, to verify whether tactile sensitivity was affected by non-informative vision, we applied one Wilcoxon signed-rank test to the detection thresholds with condition as within-subject factor.

Mean numerosity judgment accuracy, signed error and response times were calculated for each number of active electrodes and each condition. To test whether non-informative vision of the stimulated body part modulated the judgment of numerosity, we applied Wilcoxon tests to accuracy, signed error and response time with the condition as within subjects’ factor. To examine how the level of difficulty modulated the subjects’ performance, we applied Friedman tests to accuracy, signed error and response time with numerosity as within subjects’ factor.

To investigate the interaction between the two factors, number of active electrodes and condition, we ran six Wilcoxon tests, one for each number of electrodes activated with the condition as within-subject factor.

Finally, we correlated numerosity judgment accuracy and response time computing a Spearman’s correlation coefficient for each condition. This was done also to find out whether participants applied some speed–accuracy tradeoff.

Moreover, to evaluate the strength of the obtained results in terms of the magnitude of the difference in the means scores of the groups, we estimated the effect size r for each Wilcoxon signed-rank test using the formula $r = \frac{z}{\sqrt n }$. As for the interpretation of the effect sizes, we followed Cohen (Cohen J. 1988). According to his guidelines, small, medium, and large effects correspond to *r* > 0.1, *r* > 0.3, and *r* > 0.5, respectively.

Statistical analysis was conducted in Python (Python Software Foundation). The threshold for the statistical significance was set to *p* < 0.05. Whenever required, we applied false discovery rate (FDR) corrections for multiple comparisons following the Benjamini–Hochberg methods (Benjamini and Hochberg 1995; Glickman et al. 2014).

## Results

### Tactile detection threshold

**Fig. 3:**
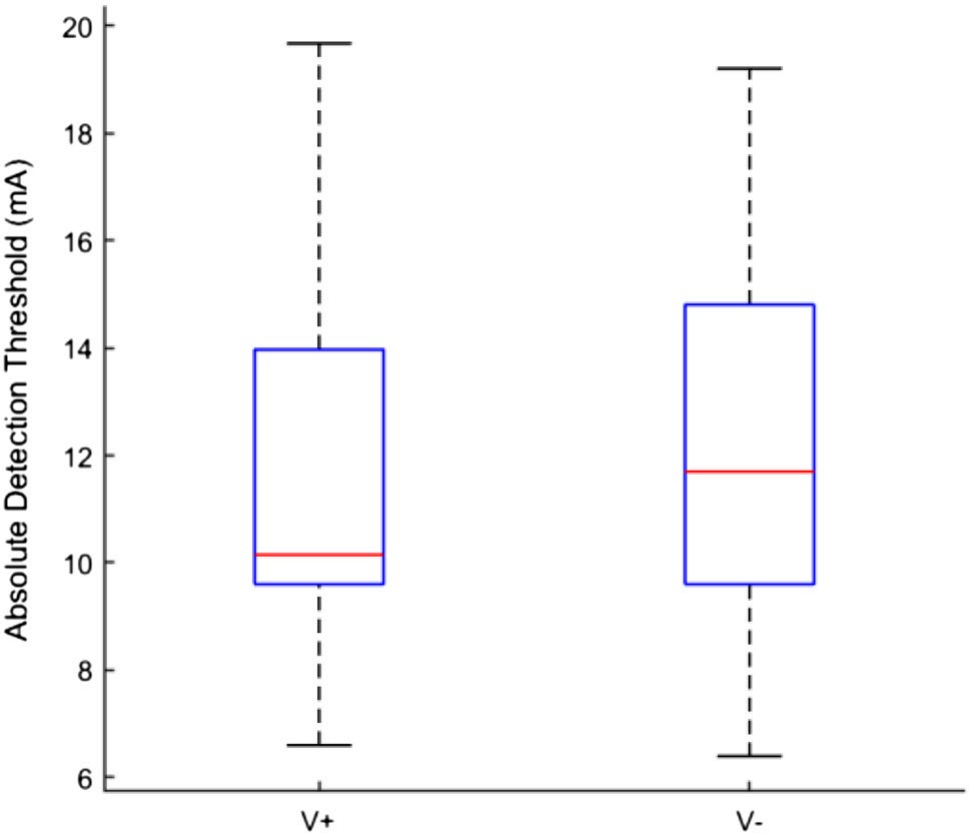
Boxplots, showing medians and 25 and 75 percentiles of the tactile detection threshold (mA). The data are grouped by condition

Results showed no differences between V + and V− in detection thresholds (V + = 11.5 ± 3.6 mA, V− = 12.2 ± 3.9 mA; T = 15, *p* = 0.20; see Fig. 3).

### Numerosity judgment accuracy

**Fig. 4:**
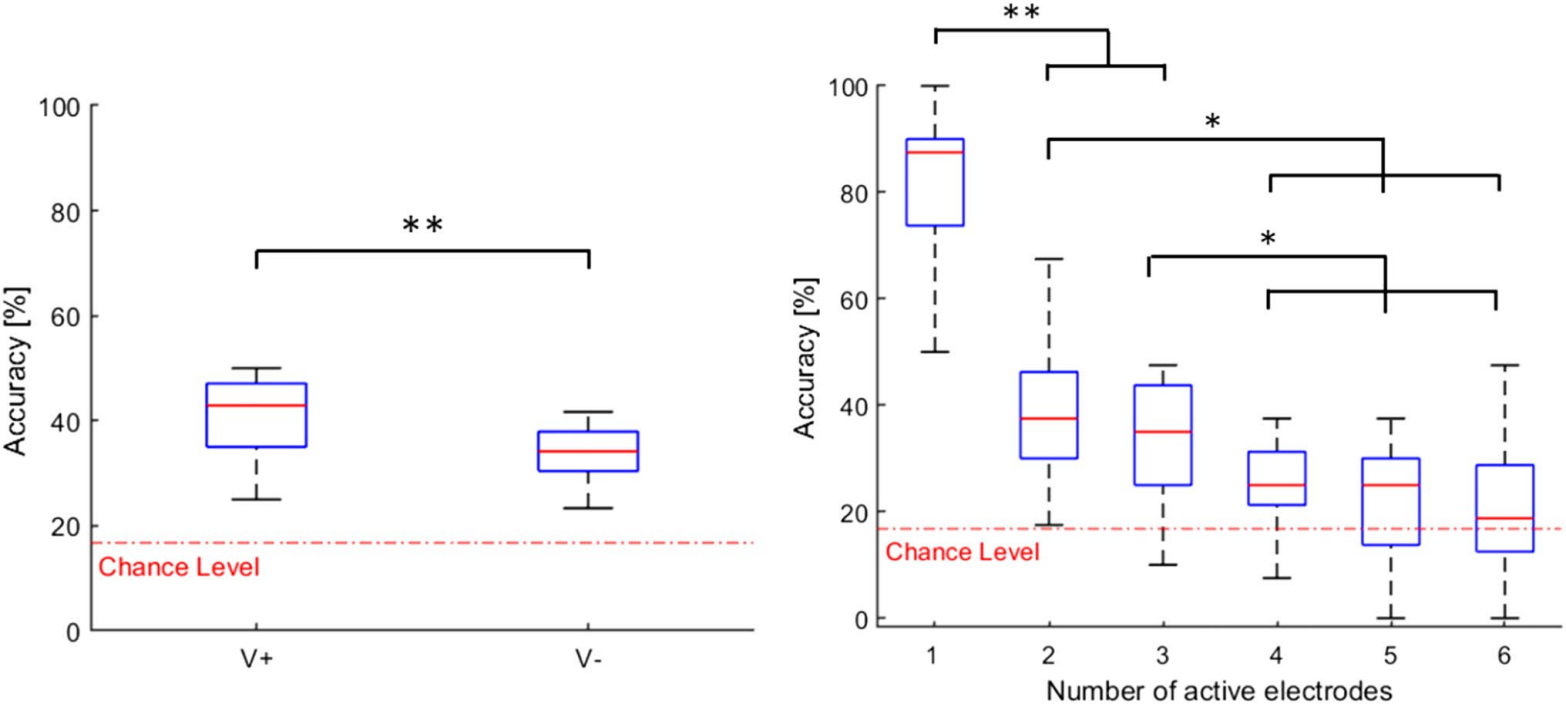
*Left panel* boxplots showing medians and 25 and 75 percentiles of accuracy for the two conditions (V + and V−). *Right panel* boxplots showing medians and 25 and 75 percentiles of accuracy per number of active electrodes. The dotted red line represents the chance level (16.7%). Asterisks indicate significant differences. *p < 0.05; **p <0.01

Accuracy data were averaged across electrodes number and submitted to a Wilcoxon test with the condition as a factor. Results showed a significant effect of the visual condition on accuracy (T = 1.5, *p* = 0.0032, *r *= 0.76). Particularly, accuracy was significantly higher in V + (40.5 ± 8%) than V− (33.8 ± 5%, see Fig. 4 left panel).

Similarly, accuracy data were averaged across the two conditions and submitted to a Friedman test with numerosity (six levels: from 1 to 6) as factor. The analysis revealed a significant effect of numerosity on accuracy (*χ*^2^ = 37.5, *p* < 0.001). Particularly, accuracy decreased as the number of active electrodes (i.e. level of difficulty) increased. Post hoc analyses showed higher accuracy when 1 electrode was activate compared to all the other levels of numerosity (*p* < 0.01, *r* > 0.9 for all cases). Moreover, accuracy when using 2 or 3 electrodes was significantly higher than when 4 to 6 electrodes were activated (both ps < 0.05, *r* > 0.7 and *r* > 0.6, respectively). On the contrary, no significant differences emerged when the number of active electrodes was 4, 5 or 6 (all ps > 0.05, see Fig. 4 right panel).

**Fig. 5:**
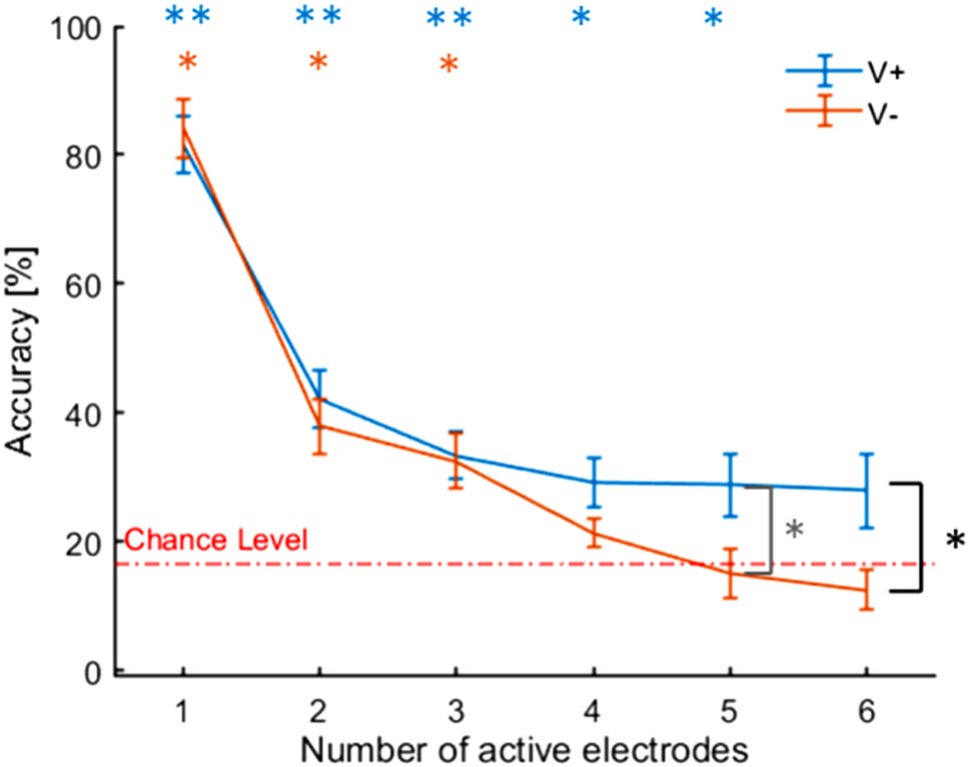
Mean accuracy per number of active electrodes (standard errors are showed) for the two conditions (V + and V−). Asterisks point to significant difference between V + e V− after FDR correction (black), between V + and V− before FDR correction (grey), between V + and chance level (light blue) and between V− and chance level (orange). *p <* 0*.05; **p <* 0*.01

Results on the interaction between condition and electrode number showed significantly higher accuracy in V + (27.9 ± 6%) compared to V− (12.5 ± 3%) when six electrodes were activated (T = 0.0, uncorrected *p* = 0.0076, FDR-corrected, *p* = 0.045, *r* = 0.69) and a trend for five active electrodes (T = 9, uncorrected *p* = 0.03, FDR-corrected *p* = 0.09). In addition, we compared accuracy and chance level (i.e., one out of six or 16.7%) separately for both conditions using six Wilcoxon tests (one for each level of numerosity). Results showed that accuracy in V + was significantly higher than chance level whenever 1 to 5 electrodes were activated (*p* < 0.05, *r* > 0.8). On the contrary, in V− this occurred only up to three electrodes (*p* < 0.05, *r* > 0.8) (see Fig. 5).

### Effect of electrode distance

**Fig. 6:**
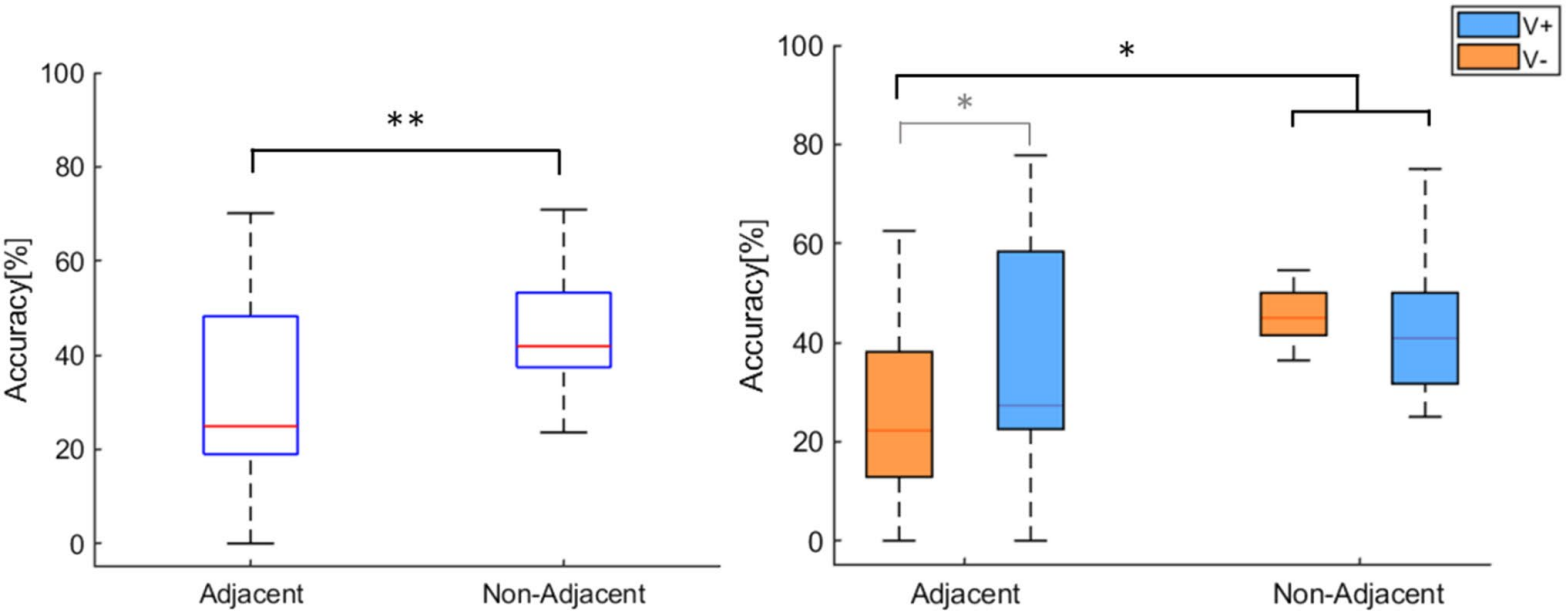
*Left panel* boxplots showing medians and 25 and 75 percentiles of accuracy for the pair configurations (adjacent and non-adjacent). *Right panel* boxplots showing medians and 25 and 75 percentiles of accuracy. Data are split for the pair configurations (adjacent and non-adjacent).The two visual conditions (V + and V−) are color-coded. Asterisks indicate significant differences (grey: before FDR correction, black: after FDR correction). *p < 0.05; **p <* 0*.01

The previous analyses did not consider the features of the randomly activated patterns of electrodes. In other words, a stimulation pattern might have included adjacent, non-adjacent electrodes or a combination of both. It is possible that the absence of effects in accuracy when few (e.g. 2) electrodes were active was due to the average of close and distant pairs which would result in a collective null result. Hence, we run an additional analysis comparing accuracy for pairs formed only by adjacent electrodes with accuracy for pairs formed by non-adjacent electrodes. In particular, we defined as non-adjacent all pairs of electrodes that included 1 or more electrodes in between (~ 10 cm) (e.g. 1-3, 2-4, 1-6, etc.) and as adjacent all pairs without intermingled electrodes (e.g. 1-2, 2-3, 3-4, etc.). We only considered pairs because, in our setup, it is not possible to select enough pure adjacent or non-adjacent configurations when three or more electrodes are active. Then we compared mean accuracy in discriminating the number of electrodes for adjacent versus non-adjacent pairs using a Wilcoxon test. Results showed a significantly higher accuracy in discriminating non-adjacent (44.7 ± 14%) than adjacent pairs (31.8 ± 20%) (*p* = 0.007, *r* = 0.7, see left panel of Fig. 6). Importantly, further analyses showed significantly higher accuracy in V + than V− when adjacent pairs were activated (uncorrected *p* = 0.026, FDR-corrected *p* = 0.052). On the contrary, these two conditions did not differ when considering non-adjacent pairs (see right panel of Fig. 6).

### Signed error

**Fig. 7:**
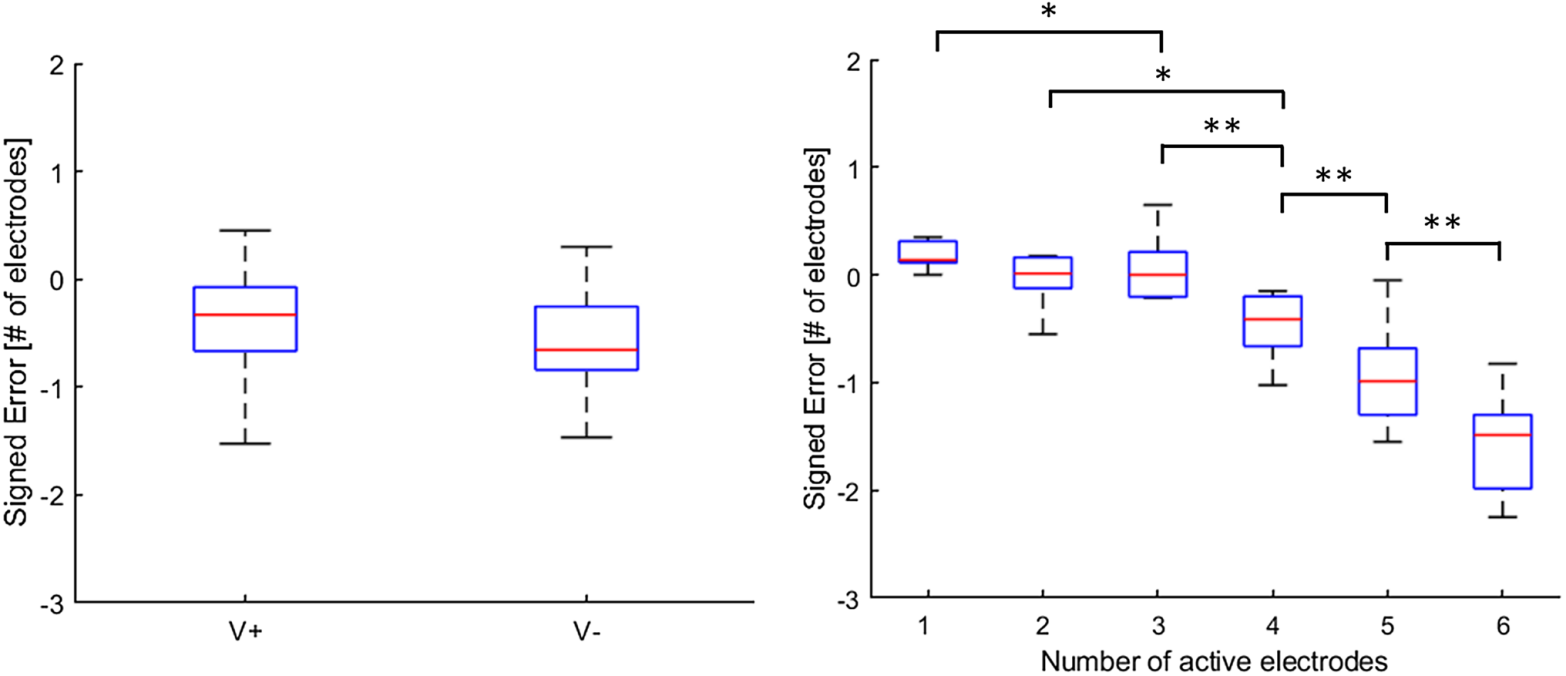
*Left panel* boxplots showing medians and 25 and 75 percentiles of signed error for the two conditions (V + and V−). *Right panel* boxplots showing medians and 25 and 75 percentiles of signed error per number of active electrodes. Asterisks indicate significant differences. *p < 0.05;**p < 0.01

Signed error data were submitted to a Friedman test with numerosity as factor. Results showed significant main effect (χ2 = 53.6, *p* < 0.001). In other words, participants strongly underestimated the number of active electrodes and the underestimation increased with the number of active electrodes. Post hoc analyses showed that the signed error at each level of numerosity differed significantly from all the others (all ps < 0.05 and *r* > 0.7 for all cases) except for when the number of active electrodes was 1, 2 or 3 (ps > 0.05). On the other hand, the signed error was not significantly affected by the condition (*p* = 0.09, see Fig. 7).

### Response time

**Fig. 8:**
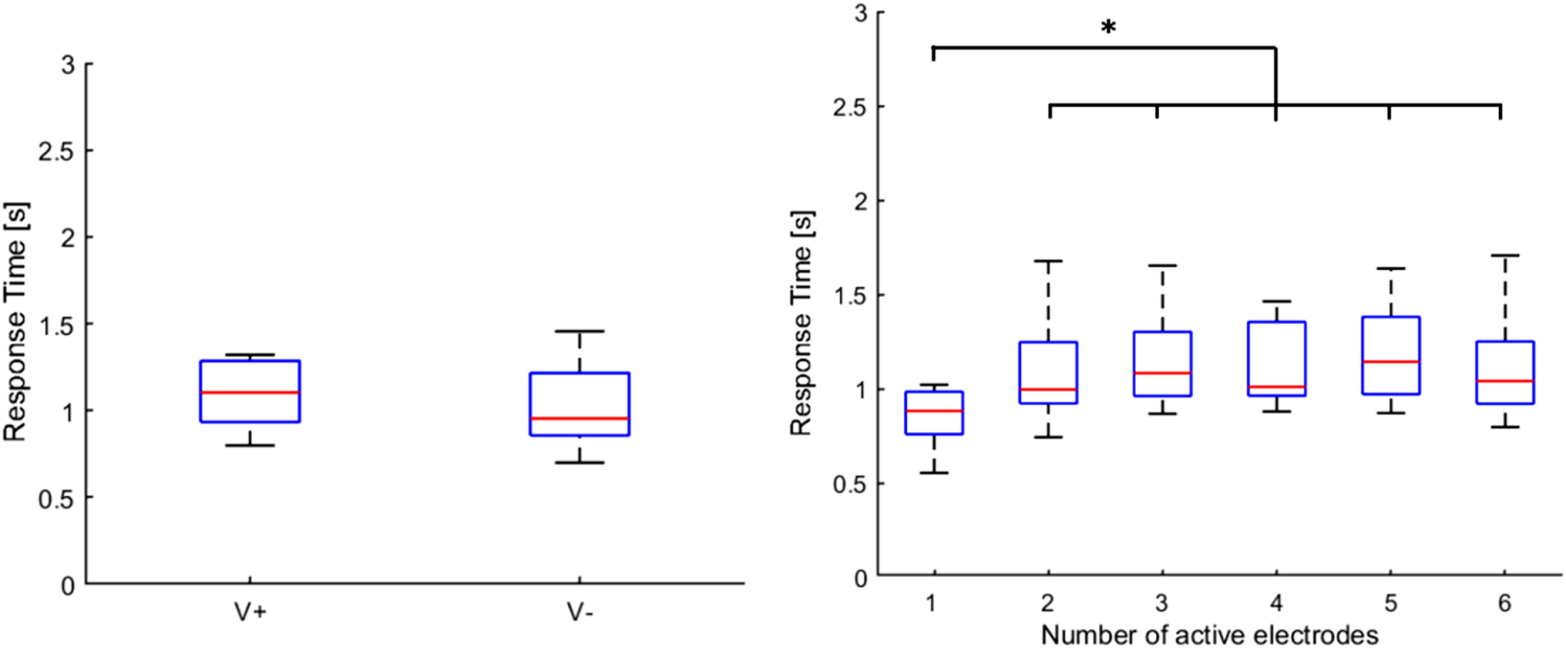
*Left panel* boxplots showing medians and 25 and 75 percentiles of response time for the two conditions (V + and V−). *Right panel* boxplots showing medians and 25 and 75 percentiles of response time per number of active electrodes. Asterisks indicate significant differences. *p < 0.05

Response times did not differ significantly between V + and V− . Response time data were also submitted to a Friedman test with combination as factor. The analysis revealed a main effect of the number of active electrodes (χ2 = 22.3, *p* < 0.001). Specifically, post hoc tests showed significantly faster subjects’ response when 1 electrode was active compared to all the other combinations (all ps < 0.05 and *r* > 0.75 for all cases, see Fig. 8).

### Correlation between response time and numerosity judgment accuracy

The Spearman correlation between response times and accuracy, separately performed for the two conditions, indicated negative non-significant correlation between these two variables (V + : *r* = − 0.223, *p* = 0.06; V− : *r* = − 0.18, *p* = 0.12). In other words, participants were neither faster nor slower when they gave a correct answer.

## Discussion

In this study, we tested whether non-informative vision of the own shoulder improves numerosity judgment and tactile sensitivity. To do so, participants performed a tactile detection threshold estimation task which measures sensitivity followed by a tactile numerosity judgment task. They performed the two tasks while watching a real-time video of their shoulder or simply a fixation cross, in counterbalanced order. Importantly, visual feedback was not informative of the timing and number of stimuli.

Results showed that visual feedback improved tactile numerosity judgment and not tactile detection threshold. This new psychophysical result adds to a substantial body of existing behavioral evidence that non-informative vision enhances tactile spatial acuity in grating orientation discrimination, two-point discrimination, vibration discrimination, proximal/distal spatial discrimination (Kennett et al. 2001; Press et al. 2004; Taylor-Clarke et al. 2004; Harris et al. 2007; Serino et al. 2007, 2009; Haggard et al. 2007; Cardini et al. 2011, 2012; Catley et al. 2014). A numerosity judgment task with electrotactile stimuli has never been used in a study investigating the visual enhancement of touch. It permits (1) to abolish any temporal cue about the stimulation (e.g. the experimenters’ hand who approaches the subjects’ hand); (2) to collect response times; (3) a factorial manipulation of the difficulty of the task, by increasing the number of simultaneous stimuli; (4) to measure eventual differences in performance between stimulation patterns formed by close or distant tactile stimuli.

Interestingly, in our numerosity judgment task, the effect of non-informative vision was initially evident only when four or more stimuli were delivered, that is when the task was more difficult and the underestimation of the number of stimuli was stronger. This result is consistent with previous findings indicating that one of the conditions to observe the visual enhancement of touch is using a task close to the limits of performance (Press et al. 2004) or testing subjects with poor tactile spatial acuity (Serino et al. 2007). However, a further analysis we performed to take into account for the distance between active electrodes showed that vision of the shoulder modulated numerosity judgment already when only two electrodes were activated but it did so only if the two electrodes were adjacent. In other words, non-informative vision enhanced the judgment of numerosity of adjacent pairs. On the contrary, no modulation was observed if the two active electrodes were not adjacent. This result is consistent with the view that the visual enhancement of touch reduces the size of tactile receptive fields. This effect might be mediated by bimodal visuotactile neurons that sharp tactile receptive fields in an early somatosensory map. These cells might be part of a corticocortical network from multimodal areas such as posterior parietal cortex and prefrontal cortex which could tune the somatosensory map of the primary somatosensory cortex to decrease the size of receptive fields, probably by increasing lateral inhibition (Kennett et al. 2001; Press et al. 2004; Haggard et al. 2007). The existence of such a network in humans has been indirectly confirmed by a couple of neurophysiological studies. First, Taylor-Clarke et al. (2002) showed a visual modulation of an N80 component localized to the primary somatosensory cortex which indicates the critical role of the modulation of S1 activity by the visual input. Similarly, Fiorio and Haggard (2005) observed a disruption of the visual enhancement of touch following a single transcranial magnetic stimulation pulse over the primary somatosensory cortex and not over the S2, highlighting the crucial role of S1 in modulating the effect. Second, Konen and Haggard (2014) have observed a similar disruption when delivering a single-pulse TMS over the anterior intraparietal sulcus which is considered crucial for integrating visual and somatosensory information related to the body (Iriki et al. 1996). The authors proposed that this brain region might provide a descending feedback signal to primary somatosensory cortex. Collectively, these findings highlight the role of feedback circuitry in multisensory interactions, that is, a view of multisensory processing which is not based only on feedforward signals from unimodal to multisensory cortex, but also on feedback signals from the multisensory to unimodal cortex (Macaluso and Driver 2005; Driver and Noesselt 2008). Another possible mechanism explaining the visuo-tactile interaction shown in the present study involves the existence of direction projections between visual and somatosensory primary areas (Cappe and Barone 2005; Ghazanfar and Schroeder 2006; Henschke et al. 2015; Teichert and Bolz 2018). The presence of visual enhancement of touch on the shoulder which is a body location normally not viewed and the behavioral correlate of the reduction of tactile receptive fields we observed hint that visuotactile neurons in humans subserving the shoulder might exist. Therefore, the frequency of viewing the body part and its involvement in exploratory movements might not be a determinant for the presence of bimodal neurons.

The proposed explanation based on receptive field size reduction in S1 mediated by visual information might also explain why the effect is specific for numerosity judgment and not non-spatial tactile sensitivity as observed in our study. Certainly, the numerosity judgment can in principle be performed based solely on the discrimination of intensity of the multiple simultaneous tactile stimuli. However, other evidences showed that the visual enhancement of touch is absent in non-spatial tasks (e.g. Press et al. 2004). Hence, we hypothesize that participants performed the numerosity judgment at least in part based on spatial discrimination. This idea is supported also by the fact that non-adjacent pairs of electrodes were better discriminated than adjacent pairs. The smaller the receptive fields, the more likely it is that a receptive field contains only one stimulus, thereby contributing to avoid the overlap between the two or more activated neuronal populations (Fuchs and Drown 1984). The reduction in the size of receptive fields would be clearly more beneficial for more complex spatial discriminations which likely contain adjacent electrodes. Collectively, we indeed observed an effect of visual feedback only when four or more stimuli were delivered at the same time.

Notably, differently than in previous studies (e.g. Press et al. 2004), we did not observe a facilitation in response times in the “view shoulder” condition. On the contrary, we observed a trend towards slower response times in the visual feedback condition. This might be due to the different task demands of the two studies. While Press and co-authors opted for a speeded spatial discrimination task, we stressed response accuracy rather than response speed. Our study differs from Press et al. study also for the kind of control condition which was a “view fixation” in the former and a “view object” in the latter. The more complex visual information in the “view shoulder” might have increased the response times compared to the “view fixation” in our study. Our results are likely not due to a speed–accuracy tradeoff as accuracy and response times did not correlate neither in the “view shoulder” nor in the “view fixation” condition.

Our results point to some possible applications for clinical rehabilitation. For instance, they suggest that visual feedback might be used as an alternative way to improve somatosensory performance in case of brain damage, as in stroke patients (Serino et al. 2007). Back pain and chronic hand pain patients provide already evidences about the effectiveness of visual feedback. For instance, it has been shown that tactile training with vision improves tactile acuity and reduces back pain (Wand et al. 2011) and chronic hand pain (Moseley and Wiech 2009). The choice of the shoulder as testing location, although there are certainly body locations less visible, also foresees these potential clinical applications (Nataletti et al. 2020). Proximal areas in the limbs are known to be less affected by sensory deficits than distal areas in stroke patients. Furthermore, the shoulder provides enough space to distribute the electrodes to obtain anatomically congruent representation of a hand. Finally, this body part is readily accessible, does not obstruct any important function and can be easily hidden under clothing.

In summary, our study adds knowledge about the role of non-informative vision in cross-modal integration that is triggered in spatial tasks. We showed that non-informative visual feedback improved spatial discrimination also in the shoulder which is a body part normally not viewed. It also confirmed that the effect is specific for tasks involving spatial acuity and not sensitivity. Finally, the mechanism mediating this effect is the reduction of the size of tactile receptive fields.

## Data Availability

The datasets used and analyzed during the current study are available from the corresponding author on reasonable request.
